# Icariside II Effectively Reduces Spatial Learning and Memory Impairments in Alzheimer’s Disease Model Mice Targeting Beta-Amyloid Production

**DOI:** 10.3389/fphar.2017.00106

**Published:** 2017-03-08

**Authors:** Lingli Yan, Yuanyuan Deng, Jianmei Gao, Yuangui Liu, Fei Li, Jingshan Shi, Qihai Gong

**Affiliations:** ^1^Department of Pharmacology and Key Laboratory of Basic Pharmacology of Ministry of Education, Zunyi Medical UniversityZunyi, China; ^2^Department of Pharmacy, Zunyi Medical UniversityZunyi, China

**Keywords:** icariside II, Alzheimer’s disease, beta-amyloid, a disintegrin and metalloproteinase domain 10, beta-site APP cleavage enzyme 1, peroxisome proliferator-activated receptor-γ

## Abstract

Icariside II (ICS II) is a broad-spectrum anti-cancer natural compound extracted from *Herba Epimedii* Maxim. Recently, the role of ICS II has been investigated in central nervous system, especially have a neuroprotective effect in Alzheimer’s disease (AD). In this study, we attempted to investigate the effects of ICS II, on cognitive deficits and beta-amyloid (Aβ) production in APPswe/PS1dE9 (APP/PS1) double transgenic mice. It was found that chronic ICS II administrated not only effectively ameliorated cognitive function deficits, but also inhibited neuronal degeneration and reduced the formation of plaque burden. ICS II significantly suppressed Aβ production *via* promoting non-amyloidogenic APP cleavage process by up-regulating a disintegrin and metalloproteinase domain 10 (ADAM10) expression, inhibited amyloidogenic APP processing pathway by down-regulating amyloid precursor protein (APP) and β-site amyloid precursor protein cleavage enzyme 1 (BACE1) expression in APP/PS1 transgenic mice. Meanwhile, ICS II attenuated peroxisome proliferator-activated receptor-γ (PPARγ) degradation as well as inhibition of eukaryotic initiation factor α phosphorylation (p-eIF2α) and PKR endoplasmic reticulum regulating kinase phosphorylation (p-PERK). Moreover, phosphodiesterase type 5 inhibitors (PDE5-Is) have recently emerged as a possible therapeutic target for cognitive enhancement *via* inhibiting Aβ levels, and we also found that ICS II markedly decreased phosphodiesterase-5A (PDE5A) expression. In conclusion, the present study demonstrates that ICS II could attenuate spatial learning and memory impairments in APP/PS1 transgenic mice. This protection appears to be due to the increased ADAM10 expression and decreased expression of both APP and BACE1, resulting in inhibition of Aβ production in the hippocampus and cortex. Inhibition of PPARγ degradation and PERK/eIF2α phosphorylation are involved in the course, therefore suggesting that ICS II might be a promising potential compound for the treatment of AD.

## Introduction

Alzheimer’s disease is the most common age-related neurodegenerative disorder. Its primary clinical symptom is progressive cognitive function deterioration, such as impaired communication, confusion, and poor judgment (Alzheimer’s Association, 2015). It is estimated that as many as 46.8 million people worldwide are suffering from dementia in 2015 and the number of AD patients rises by more than 11 million people per year, which leads to a tremendous burden on the society and the family (Carrillo et al., 2013; Alzheimer’s Association, 2015). Clinically, the hallmarks of AD include the deposition of senile plaques, which are comprised of Aβ peptides neurofibrillary tangles (NFTs), and the death of neurons accompanied by synapses loss (Yang R.Y. et al., 2015). Although the pathogenesis of AD is complicated and the mechanisms governing this disease remains elusive, extensive studies suggest that Aβ cascade hypothesis remains dominating the AD research, which centers on Aβ as the most critical initiator in the pathogenesis of AD (Sun et al., 2015; Sengupta et al., 2016). Aβ results from the sequential proteolytic processing of APP by BACE1 (as the rate-limiting secretase) and γ-secretase, the cleavage step is contributes much to AD pathology (Zhang and Song, 2013). However, in the non-amyloidogenic cleavage process, APP is cleaved by α-secretase (a large metallopeptidase family, known as ADAM) to produce non-toxic fragments, which is deemed to antagonize Aβ generation (Vassar, 2013; Sun et al., 2015). Excessive accumulation and aggregation of Aβ result in neurotoxicity in the nervous system, inducing neuronal degeneration and eventually the death of neurons (Zhu et al., 2013), which further causes memory deficits (Sun et al., 2015). Currently clinical available pharmacotherapies of AD include acetylcholinesterase inhibitors (such as donepezil, tacrine, rivastigmine, and galantamine) and *N*-methyl-D-aspartate receptor antagonists (memantine) (Fan and Chiu, 2014). Given that the etiology of AD is multiple, these agents only ameliorate certain symptoms but do not change or delay the progress of AD which substantially limits their clinical utilities (Anand et al., 2013). Thus, new therapies for AD are in dire need.

Among numerous therapeutic strategies of AD, BACE1 has always attracted much attention to investigate it’s biological function on APP-Aβ process. Increasing evidence indicated that BACE1 overexpression and Aβ accumulation were observed in brains of AD mouse models and patients. However, Aβ and β-secretase-cleaved APP fragments were decreased in BACE1-knockdown model mice (Sankaranarayanan et al., 2008). Thus, the relationship between BACE1 and Aβ production has explicitly point out that screening out compound for decreasing BACE1 level for against AD. But to date, majority of compounds that inhibiting BACE1 still exist many side effects in clinical trials (including peptides and synthetic). Several natural compounds isolated from Chinese medical herbs, such as ginsenoside, because of its pharmacological safety and therapeutic potential, which are widely applied in prophylaxis and treatment of various illness (Yang X.-D. et al., 2015; Cao et al., 2016; Xie et al., 2016).

*Herba Epimedium* is a popular Chinese medicinal plant, traditionally used as cardiovascular diseases and cancer therapy (Sze et al., 2010). One of its bioactive flavonoid compounds, ICS II, shows good CNS penetration and robust biological activities. ICS II protect against cerebral ischemia reperfusion injury in rats *via* an integrated mechanism of nuclear factor-κB inhibition and peroxisome proliferator activated receptor up-regulation (Deng et al., 2016). Similarly, ICS II alleviates hippocampal injury in a gerbil model of ischemia-reperfusion (Yan et al., 2014). In addition, our previous research has shown that ICS II attenuates streptozotocin-induced cognitive deficits and neuronal degeneration, the level of Aβ contents are also decreased in rats, the underlying mechanisms of which may be associated with the inhibition of BACE1 level (Zhang and Song, 2013; Yin et al., 2016). However, it is unclear whether ICS II could ameliorate cognitive function impairments *via* inhibiting multiple pathogenic pathways of Aβ production in APP/PS1 transgenic mice.

In this study, APP/PS1 transgenic mice were utilized to examine whether chronic treatment of ICS II could improve spatial learning and ameliorate memory impairments. We also examined the underlying biochemical mechanisms related to the behavioral changes. Here we show that ICS II effectively ameliorated cognitive functions deficits *via* inhibiting neuronal loss and the formation of senile plaques, at least partly, by decreasing Aβ production.

## Materials and Methods

### Reagents

Icariside II ≥98% by (HPLC), which was provided from Nanjing Zelang Medical Technology Corporation Ltd (China). All related experiment reagents were reagent grade and commercially available.

### Animals

All male APPswe/PS1dE9 transgenic model mice (APP/PS1) and their littermates WT mice (weighing 30–35 g) were obtained from the Model Animal Research Centre of Nanjing University. Mice were allowed to acclimatize under SPF-class animal housing of laboratory (certificate no. SYXK 2011-004) at Zunyi Medical University, with 12 h light/dark cycle, temperature (22 ± 1°C), relative humidity (60–70%), food and drinking water were available *ad libitum*. Animal experiments were strictly implemented according to the State Committee of Science and Technology of the People’s Republic of China Order No. 2 on November 14, 1988 (revised 2011) and the protocols in this study were allowed by the Animal Experimentation Ethics Committee of the Zunyi Medical University.

### Animal Treatments

Nine-month-old male APP/PS1 transgenic mice were randomized divided into three groups: ICS II treated groups (10 mg/kg, *n* = 12; 30 mg/kg, *n* = 13) and APP/PS1 control group (*n* = 12). Age-matched male WT mice were randomized assigned to three groups: ICS II treated groups (10 and 30 mg/kg, *n* = 10) and WT control group (*n* = 10). APP/PS1 and WT treated groups were orally administered with ICS II dissolved in NS at a dose of 10, 30 mg/kg body weight once daily, and control groups received volume-matched NS for 3 months.

### Morris Water Maze (MWM) Test

After 3 months of treatment, MWM task was applied to evaluate cognitive abilities of the mice (under the condition of experimenters blinded to the treatments). In brief, the apparatus consisted of a circular white plastic water tank (the diameter was 120 cm and height was 50 cm) and filled with water (24 ± 1°C) to a depth of 30 cm, the area of tank was divided into four quadrants of equal size, a removable circular platform of perspex (9.5 cm diameter, 29 cm height) was hidden in the midpoint of the third quadrant and submerged 1 cm beneath the surface of water for all trials and sessions. Spatial acquisition ability was measured during four consecutive days, during which the mouse was released into the water that one of the four quadrants and allowed 60 s to reach the hidden platform and keep on it for 20 s, and the escape latency (swim toward the hidden platform and keep on it for more than 3 s) were recorded. If mice failed to found the hidden platform within preset time (the escape latency was recorded as 60 s), and it would be softly guided to the hidden platform where it remained on top of the platform for 20 s. Per mouse was experimented to four trials per day and the inter-trial interval was 20 min. A probe trial was performed in the fifth day to measure the final spatial memory consolidation (hidden platform was withdrawn). The percentage of time of each mouse was recorded during a 60 s trial. These activities in all trials were automatically measured and analyzed by using the behavior analyzing system (TopScan Version 3.00).

### Tissue Preparation

All animals were deeply anesthetized with sodium pentobarbital (50 mg/kg intraperitoneally) after MWM test, and mice (*n* = 4–5 per group) were perfused transcardially with ice-cold 0.01M phosphate-buffered saline (PBS, pH = 7.4), followed by the precooled 4% buffered paraformaldehyde (pH = 7.4), then the brain was instantly removed and stored in fresh fixative at 4°C overnight. The right hemispheres of brains were dehydrated through the gradient of 20 and 30% sucrose solutions until sank at 4°C. The respective left hemispheres were embedded by paraffin. For other animals (*n* = 6–8 per group), the hippocampus and cortex were immediately isolated and stored at -80°C with Eppendorf micro test tubes.

### Thioflavine S and Nissl Staining

The right hemispheres of brains were serially cut using a cryostat (Leica CM 1850 UV; Leica, Nussloch, Germany) at 30 μm thickness sections in the coronal plane. The brain slices were collected sequentially in 24-well plates, which filling with anti-freezing solution and stored at -20°C. To observe the formation of senile plaques in hippocampus of mice, the brain slices were placed in a 1% thioflavine S (Lot# SLBG4212V, Sigma, USA) for 10 min, and then eluted with 70% alcohol, and finally washed with PBS three times and mounted with glycerin jelly, with all of the steps carefully kept away from light. The green fluorescence-tagged senile plaques were examined under a fluorescence microscope (BX53+DP80, Olympus, Japan). The numbers of senile plaques in the hippocampus were counted by Image-Pro Plus (Media Cybernetics, Bethesda, MD, USA). Meanwhile, the left hemispheres of brains fixed with fresh 4% paraformaldehyde solution for 1 week at 4°C, Finally, the samples were embedded in paraffin. Three-micrometer-thick brain tissue coronal sections of mice were stained with toluidine blue (Solarbio, China). The Nissl bodies were stained blue-purple in the CA3 and DG regions of hippocampus. The morphological changes (normal neurons having granular cytoplasm and euchromatic nucleus with large nucleoli) were observed under a light microscope (KS300, Zeiss-Kontron, Germany). Viable neurons in the CA3 and DG region of hippocampus from each group were counted as previously described (Liu et al., 2015).

### Western Blot Analysis

Hippocampus and cortex tissues (*n* = 3–4 per group) were homogenized at 4°C in the radio-immunoprecipitation (RAPI) assay lysis buffer containing complete protease inhibitor mixture by using a plastic homogenizer and then sonicated for 30 min on an ice plate. The homogenized tissue were centrifuged for 15 min (12000 × *g*, 4°C), and supernatant was extracted and subpackaged, finally stored at -80°C. The concentrations of total protein were tested by BCA protein assay kit (Beyotime, China), and equal amounts of protein (approximately 25 or 30 μg) were heat-denatured at 100°C for 5 min, electrophoretically separated using 6–12% SDS-PAGE gels, electrophoretic transferred onto the PVDF membranes (0.45 μm). Membranes were then blocked with 2% BSA or 5% defatted in 1 × TBST for 1–3 h and next probed with corresponding primary antibodies against APP (1:1,000, AB60097b, BBB Life sciences, USA), Aβ_1-40_ (1:1,000, MAB2675, Abnova, USA), Aβ_1-42_ (1:1,000, #14974, CST, USA), PDE5A1 (1:1,000, ab14672, Abcam, USA), sAPPα (2B3) (1:500, Immuno-Biological Laboratories, USA), ADAM10 (1:500, ab124695, Abcam, USA), sAPPβ (6A1) antibody (1:500, Immuno-Biological Laboratories, USA), BACE1 (1:1,000, ab108394, Abcam, USA), PPARγ (1:500, ab19481, Abcam, USA), p-PERK (Thr981) (1:200, sc-32577, Santa Cruz Biotechnology, USA), total PERK (1:200, sc-13073, Santa Cruz Biotechnology, USA), p-eIF2α (Ser51) (1:200, ab32157, Abcam, USA), total eIF2α (1:500, ab5369, Abcam, USA), and β-actin (1:2,000, AA128, Beyotime, China) overnight at 4°C. After incubation with appropriate HRP-conjugated secondary antibodies for 1–2 h at room temperature, the immunoreactive protein was exhibited with ECL detection reagent (Beyotime, China) and the densitometry of band was analyzed using Quantity One-4.6.7. (Bio-Rad, USA).

### Statistics

The data were presented as mean ± SEM. All statistical analyses were performed using SPSS software, version 17.0 (SPSS, Chicago, IL, USA). MWM task data were analyzed using repeated measures analysis of variance (ANOVA). Firstly, Mauchly’s test of sphericity should be used to judge whether there were relations among the repeatedly measured data. If any (*P* < 0.05), and the Greenhouse-Geisser corrected results should be taken. With multivariate ANOVA, date in different treated group of each measurement time could be compared pairwise.

For the data that did not involve repeated measures, they were analyzed using one-way ANOVA. If the ANOVA test results were significant, the individual differences among different groups or conditions were determined by *post hoc* least significant difference (LSD). A probability value of <0.05 was statistically considered significant differences.

## Results

### Effects of ICS II on Spatial Learning and Memory in APP/PS1 Transgenic Mice

**FIGURE 1:**
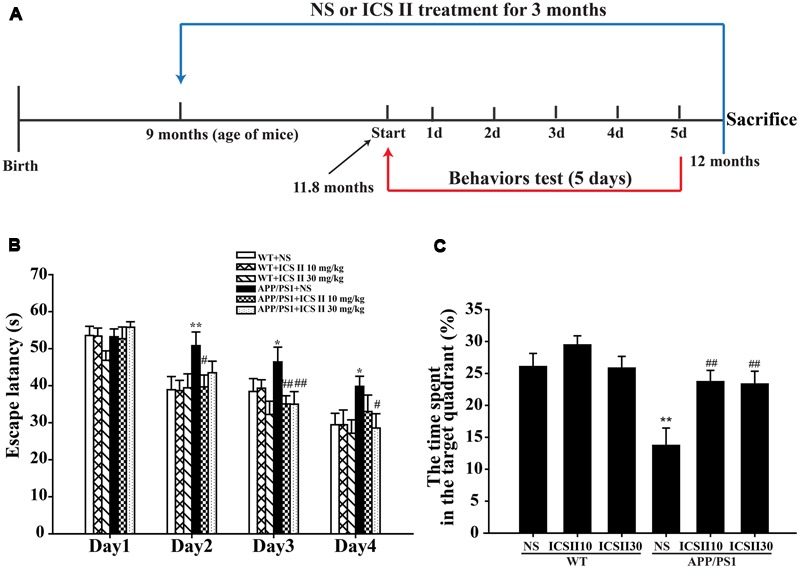
**Effects of ICS II on spatial learning and memory functions in APP/PS1 transgenic mice. (A)** The flow chart of animal experiments. **(B)** The escape latency of mice to reach the hidden platform from day 1 to day 4. **(C)** The percent of time that mice swimming in the target quadrant. Values were expressed as mean ± SEM (WT+NS, *n* = 9; WT+ICS II 10 mg/kg, *n* = 10; WT+ICS II 30 mg/kg, *n* = 10; APP/PS1+NS, *n* = 12; APP/PS1 + ICS II 10 mg/kg, *n* = 12; WT+ICS II 30 mg/kg, *n* = 13), ^∗^*p* < 0.05, ^∗∗^*p* < 0.01 vs. WT+NS, ^#^*p* < 0.05, ^##^*p* < 0.01 vs. APP/PS1+NS. Abbreviation: SEM, standard error of the mean; NS, normal saline.

To investigate the effects of ICS II on cognitive deficits in APP/PS1 transgenic mice, the spatial learning and memory of all mice were evaluated through the MWM. The *P*-value of Mauchly’s Test of Sphericity in escape latency was less than 0.05 (*P* = 0.017), which did not accept the sphericity assumption. Then the Greenhouse-Geisser correction was used, and the main effect of “latency between days” and “latency between groups” was significant difference [*F*_(2.619,157.127)_ = 55.309, *p* < 0.001] and [*F*_(5,60)_= 2.912, *p* = 0.02], respectively. The interaction of “days × groups” was no significant [*F*_(13.094,157.127)_= 1.520, *p* = 0.115]. During the consecutive 5 days of training, acquisition ability of spatial learning and memory were measured (**Figure 1**). In the navigation test, as illustrated in **Figure 1B**, the escape latency of all mice was gradually shortened and their ability to locate the platform was improved with the training. On days 2–4 (**Figure 1B**), APP/PS1 transgenic mice showed increased mean escape latencies (*p* < 0.05). With the chronic treatment with ICS II at the dose of 30 mg/kg, no obvious differences in mean escape latencies were observed on the first and second days compared with APP/PS1 control group, while notable differences were detected on the third and fourth days (*p* < 0.05, *p* < 0.05, respectively). Interestingly, on days 2–3, the mean escape latencies were reduced after treatment with ICS II at the dose of 10 mg/kg for 3 months (*p* < 0.05, *p* < 0.01, respectively). But there was no noticeable change on day 4 (*p* > 0.05).

The percentage of time that animals spent in the target quadrant during the search for the platform in probe trial is shown in **Figure 1C**. There was a very low percentage on time spent in target quadrant in APP/PS1 control group (*p* < 0.01) [*F*_(5,60)_ = 6.860, *p* < 0.001], indicative of cognitive function deficits. Nevertheless, ICS II (10 mg/kg) treated group spent more time in the target quadrant (*p* < 0.01). Similar effect was observed at a larger dose of ICS II (30 mg/kg) (*p* < 0.01). In addition, the swimming speed did not show significant differences among the groups, suggesting that ICS II treatment did not affect the motor ability in mice. Together, these results indicated that chronic treatment of ICS II could ameliorate the spatial learning and memory impairments in APP/PS1 transgenic mice.

### The Effects of ICS II on Neuronal Cells in the Hippocampus of APP/PS1 Transgenic Mice

**FIGURE 2:**
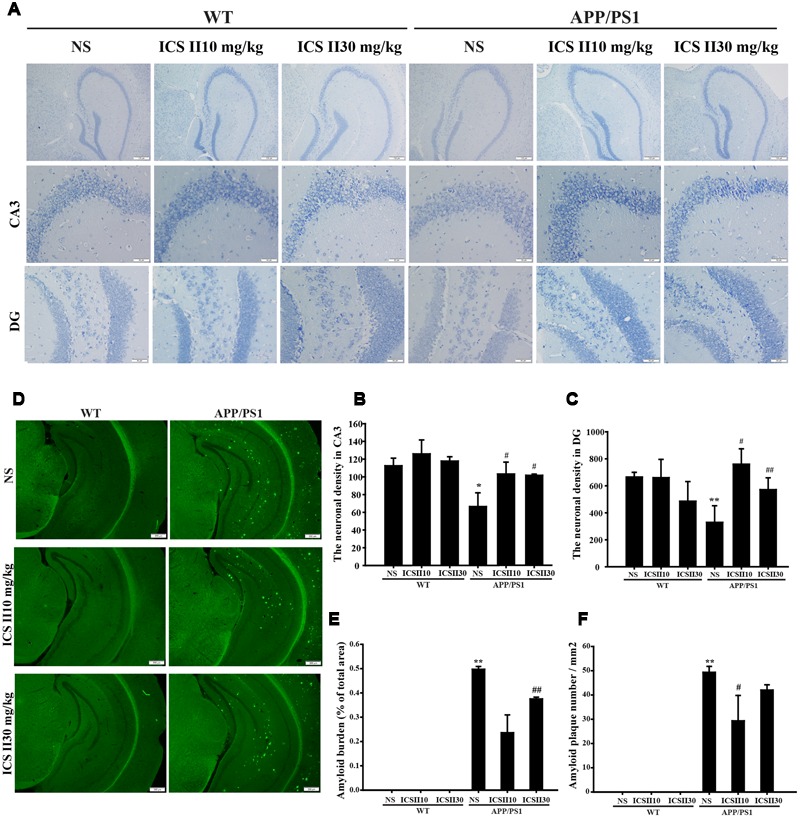
**Effects of ICS II on neuronal cells and senile plaque formation in the hippocampus of APP/PS1 transgenic mice. (A)** Representative photomicrographs of Nissl staining results of each group (magnification 400×, scale bar = 50 μm). **(B,C)** Statistics of viable neurons in the hippocampal CA3 and DG regions. **(D)** Images of thioflavine S-stained senile plaques results of each group (magnification 40×, scale bar = 200 μm). **(E,F)** Statistics of thioflavine S-stained amyloid burden and amyloid plaques number in the hippocampus. Values were expressed as mean ± SEM (*n* = 3 each), ^∗^*p* < 0.05, ^∗∗^*p* < 0.05 vs. WT+NS, ^#^*p* < 0.05, ^##^*p* < 0.01 vs. APP/PS1+NS. SEM, standard error of the mean; NS, normal saline.

The viable neuron is believed to be an especially important role in the learning and memory functions. Therefore, the number of neuronal cells in the CA3 and DG regions of hippocampus were evaluated to investigate the effects of ICS II treatment on the neuronal morphology change by Nissl staining. As the shown in **Figure 2A**, normal morphological features of pyramidal cells is intact structure, including granular cytoplasm and whereas the apparent pathological changes with loosely arranged neurons and less neurons were found in APP/PS1 transgenic mice hippocampal CA3 and DG regions, viable neurons were counted using three equally spaced coronal sections passing through the hippocampus for each brain [*F*_(5,12)_ = 3.359, *p* = 0.04; *F*_(5,12)_ = 4.975, *p* = 0.011] (*p* < 0.05; *p* < 0.01) (**Figures 2B,C**). The mean numbers of neurons were clearly more with ICS II treatment than controls (*p* < 0.05; *p* < 0.05). Overall, these data demonstrated that ICS II attenuated neuronal death in APP/PS1 transgenic mice.

### Effects of ICS II on Senile Plaque Formation in the Hippocampus of APP/PS1 Transgenic Mice

In the brain of AD patients, the most remarkable neuropathological feature is Aβ overproduction, which ultimately causes the formation of senile plaque. In the present study, we evaluated the effects of ICS II on the formation of senile plaque in coronal sections of hippocampus from different groups (**Figure 2D**). Senile plaque formation was not found in WT mice at the age of 12 months, in contrast, thioflavine S staining revealed the presence of extensive senile plaques in the hippocampus of age-matched APP/PS1 transgenic mice. Importantly, quantification analysis exhibited that the amyloid burden (% of total areas) was remarkably reversed by ICS II [*F*_(2,6)_ = 9.116, *p* = 0.015] (*p* < 0.01) (**Figure 2E**), whereas was not found difference in low dose of ICS II 10 mg/kg. Simultaneously, the number of amyloid plaque was significantly decreased in APP/PS1 transgenic mice treated with ICS II (10 mg/kg) when compared with APP/PS1 transgenic mice [*F*_(2,6)_ = 3.618, *p* = 0.093] (*p* < 0.05) (**Figure 2F**). Taken together, these results suggested an inhibitory effect of ICS II on amyloid plaque formation.

### Effects of ICS II on the Levels of Aβ_1-42_ and Aβ_1-40_ in the Hippocampus and Cortex of APP/PS1 Transgenic Mice

**FIGURE 3:**
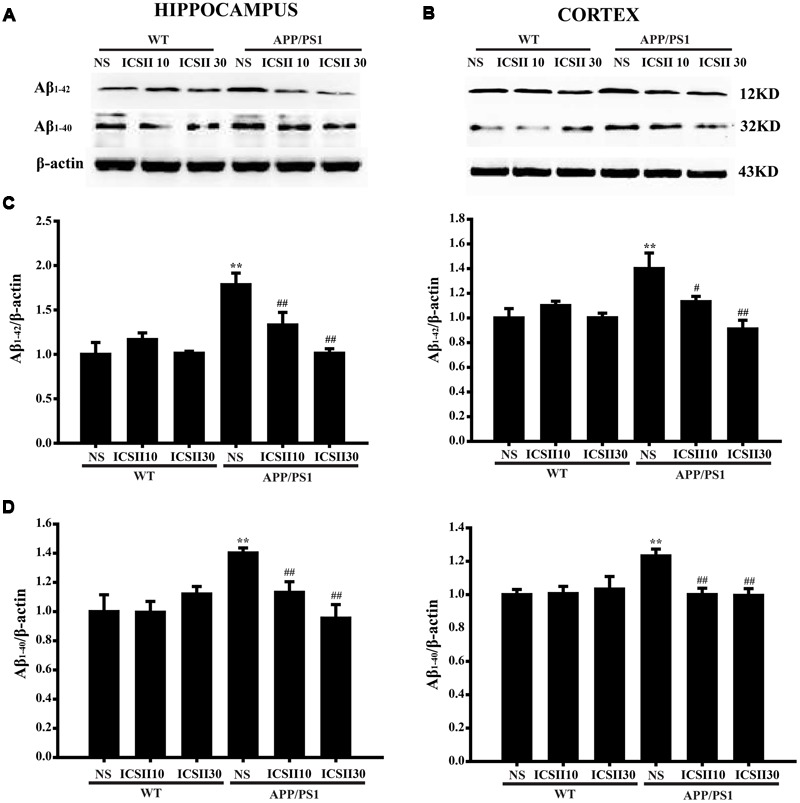
**Effect of ICS II on the levels of Aβ_1-42_ and Aβ_1-40_ in the hippocampus and cortex of APP/PS1 transgenic mice. (A,B)** The antibody-reactive bands of Aβ_1-42_ and Aβ_1-40_ in the hippocampus and cortex of different groups. **(C)** Quantitative analysis of Aβ_1-42_ levels. **(D)** Quantitative analysis of Aβ_1-40_ levels. The relative optical density was normalized to β-actin. Data are presented as mean ± SEM (*n* = 3 each), ^∗∗^*p* < 0.01 vs. WT+NS, ^#^*p* < 0.05, ^##^*p* < 0.01 vs. APP/PS1+NS. SEM, standard error of the mean; NS, normal saline; Aβ, Amyloid-β.

Since accumulating evidence indicates that AD mice exhibits accelerated Aβ production in the brain with increasing age. Thus, the levels of Aβ contents in the hippocampus and cortex of mice were subsequently examined using Western blot. As depicted in **Figures 3A–D**, the levels of Aβ_1-42_ (*p* < 0.001; *p* < 0.01) [Hippocampus (*F*_(5,12)_ = 9.007, *p* < 0.001); Cortex (*F*_(5,12)_ = 5.667, *p* < 0.01)] and Aβ_1-40_ (*p* < 0.001; *p* < 0.01) [Hippocampus (*F*_(5,12)_ = 29.012, *p* < 0.001); Cortex (*F*_(5,12)_ = 3.869, *p* = 0.025)] were markedly enhanced in APP/PS1 transgenic mice. However, after treated with two doses of ICS II resulted in a dramatic reduction in both Aβ_1-42_ and Aβ_1-40_ (*p* < 0.01; *p* < 0.05) (*p* < 0.01; *p* < 0.01). In addition, ICS II treatment in WT mice did not facilitate the Aβ levels. Together, these results indicated that long-term ICS II treatment significantly reduces Aβ_1-42_ and Aβ_1-40_ levels.

### Effects of ICS II on Process of Aβ Production in the Hippocampus and Cortex of APP/PS1 Transgenic Mice

**FIGURE 4:**
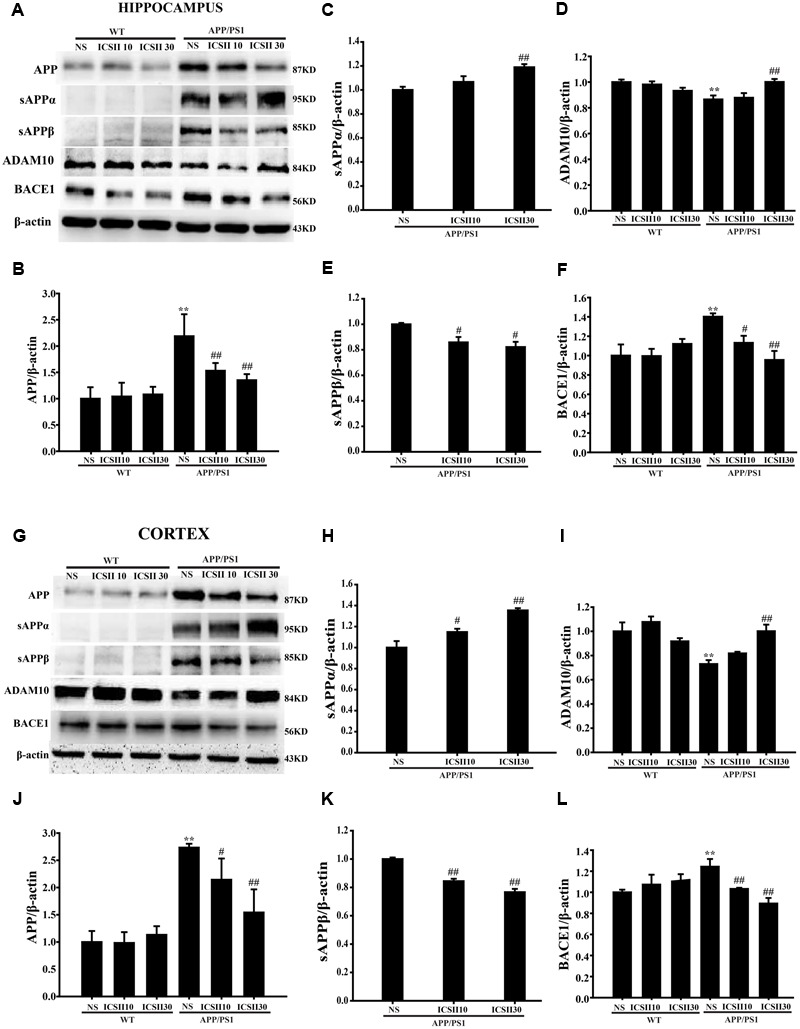
**Effect of ICS II on process of amyloid-β production in the hippocampus and cortex of APP/PS1 transgenic mice. (A,G)** The antibody-reactive bands of APP, sAPPα, and ADAM10 as well as sAPPβ and BACE1 in the hippocampus and cortex of different groups. **(B,J)** Quantitative analysis of APP protein expression. **(C,H)** Quantitative analysis of sAPPα protein expression. **(E,K)** Quantitative analysis of sAPPβ protein expression. **(D,I)** Quantitative analysis of ADAM10 protein expression. **(F,L)** Quantitative analysis of BACE1 protein expression. The relative optical density was normalized to β-actin. Data are presented as mean ± SEM (*n* = 3 each), ^∗∗^*p* < 0.01 vs. WT+NS, ^#^*p* < 0.05, ^##^*p* < 0.01 vs. APP/PS1+NS. SEM, standard error of the mean; NS, normal saline; APP, amyloid precursor protein; sAPPα, soluble APP-α; sAPPβ, soluble APP-β; BACE1, β-site APP cleavage enzyme 1; ADAM10, a disintegrin and metalloproteinase domain 10.

Abnormally increased Aβ production closely correlates with the secretases of APP cleavage process, therefore we next assessed the process of APP changes which could contributes to the decreased Aβ production. As showed in **Figures 4A–L**, quantitative analysis results indicated significant differences in the protein level of APP in APP/PS1 model group compared with WT control group (*p* < 0.01; *p* < 0.01) [Hippocampus *F*_(5,12)_ = 10.670, *p* < 0.001; Cortex *F*_(5,12)_ = 20.739, *p* < 0.001] (**Figures 4B,J**). Moreover, chronic treatment with ICS II significantly suppressed the APP level in a concentration dose-dependent manner (*p* < 0.01; *p* < 0.05). Because two key enzymes, ADAM10 and BACE1, are involved in the initial cleavage of APP, we further analyzed the ADAM10, BACE1 protein expressions and the levels of major APP-cleaved product (sAPPα and sAPPβ). Quantitative analysis showed that ICS II treatment markedly increased the level of sAPPα [Hippocampus *F*_(2,6)_ = 7.515, *p* = 0.023 and Cortex *F*_(2,6)_ = 18.272, *p* = 0.003] (*p* < 0.01; *p* < 0.01) and decreased the level of sAPPβ [Hippocampus *F*_(2,6)_ = 7.662, *p* = 0.022 and Cortex *F*_(2,6)_ = 46.559, *p* < 0.001] (*p* < 0.05; *p* < 0.01) in APP/PS1 control group. However, the dose of 10 mg/kg ICS II did not change the levels of sAPPα in the hippocampus (*p* = 0.183) and cortex (*p* = 0.242).

Moreover, in the APP/PS1 transgenic mice, the ADAM10 level was dramatically reduced relative to WT control group [Hippocampus *F*_(5,12)_ = 5.177, *p* = 0.009 and Cortex *F*_(5,12)_ = 8.221, *p* = 0.001] (*p* < 0.01; *p* < 0.01) (**Figures 4D,I**). In contrast, the protein levels of BACE1 were clearly enhanced (*p* < 0.01; *p* < 0.01) [Hippocampus *F*_(5,12)_ = 4.427, *p* < 0.05 and Cortex *F*_(5,12)_ = 9.304, *p* < 0.01] (**Figures 4F,L**). Interestingly, ICS II (30 mg/kg) could also blunt the down-regulation of ADAM10 (*p* < 0.01; *p* < 0.01). ICS II (10, 30 mg/kg) significantly down-regulated the BACE1 protein levels (*p* < 0.05; *p* < 0.01) and no differences were found among the WT animals. These results may suggest that ICS II inhibited Aβ production *via* reducing the protein expression of APP and BACE1 and inducing ADAM10 protein expression in APP/PS1 transgenic mice.

### The Inhibition of eIF2α and PERK Phosphorylation Mediated Pathway and Suppression of PPARγ Degradation Were Involved in the ICS II-Reduced BACE1 Effect in APP/PS1 Transgenic Mice

**FIGURE 5:**
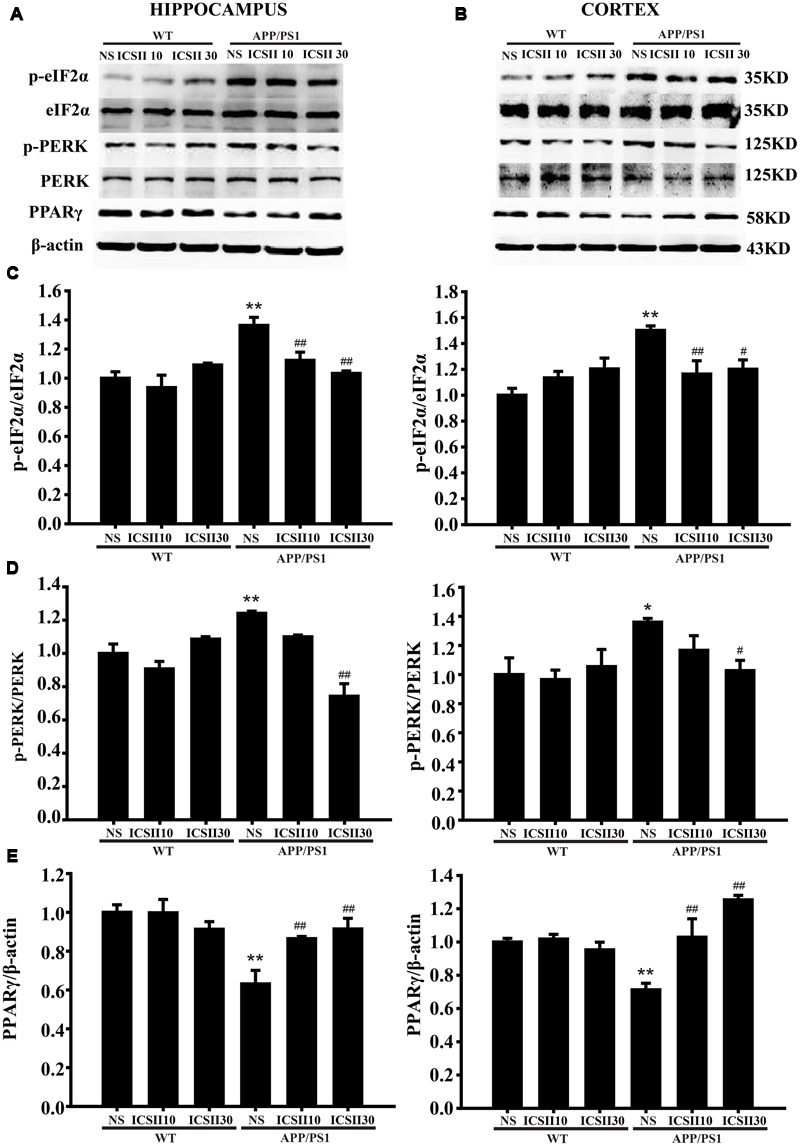
**Effects of ICS II on the PPARγ expression and the phosphorylation levels of eIF2α and PERK in APP/PS1 transgenic mice. (A,B)** The antibody-reactive bands of p-eIF2α, p-PERK, and PPARγ in the hippocampus and cortex of different groups. **(C)** Quantitative analysis of p-eIF2α levels. **(D)** Quantitative analysis of p-PERK levels. The relative optical density was normalized to eIF2α and PERK. **(E)** Quantitative analysis of PPARγ protein expression. The relative optical density was normalized to β-actin. Data are presented as mean ± SEM (*n* = 3 each), ^∗^*p* < 0.05, ^∗∗^*p* < 0.01 vs. WT+NS, ^#^*p* < 0.05, ^##^*p* < 0.01 vs. APP/PS1+NS. SEM, standard error of the mean; NS, normal saline; eIF2α, eukaryotic initiation factor α; PERK, PKR endoplasmic reticulum regulating kinase; PPARγ, peroxisome proliferator-activated receptor-γ.

Phosphorylation eIF2α/PERK and PPARγ are important signaling pathway could regulate BACE1 levels. Thus, we next examined whether ICS II could inhibited the level of p-eIF2α or p-PERK in the brains of APP/PS1 transgenic mice (**Figures 5A,B**). The levels of p-eIF2α[Hippocampus *F*_(5,12)_ = 5.632, *p* = 0.007; Cortex *F*_(5,12)_ = 5.597, *p* = 0.007] (*p* < 0.01; *p* < 0.001) and p-PERK (*p* < 0.01; *p* < 0.05) [Hippocampus *F*_(5,12)_ = 10.561, *p* < 0.001; Cortex *F*_(5,12)_ = 2.743, *p* = 0.071] in APP/PS1 transgenic mice both were much higher than WT control group (**Figures 5C,D**), which is in agreement with previous studies and also consistent with the results of BACE1 level. Chronic ICS II (30 mg/kg) treatment significantly inhibited the levels of p-eIF2α (*p* < 0.01; *p* < 0.05) and p-PERK (*p* < 0.01; *p* < 0.05). The results further support the notion that ICS II has neuroprotective effect in APP/PS1 transgenic mice and that the mechanisms may involve the reduction of BACE1 *via* dephosphorylation of eIF2α/PERK. Another transcription factor, PPARγ, can regulate BACE1 activity and reduce Aβ production. Here, we also examined the PPARγ level in the hippocampus and cortex of APP/PS1 transgenic mice (**Figures 5A,B**). As compared to WT control group, APP/PS1 transgenic mice exhibited a lower level of PPARγ in different brain regions (*p* < 0.01; *p* < 0.01) [Hippocampus *F*_(5,12)_ = 7.045, *p* = 0.003; Cortex *F*_(5,12)_ = 10.064, *p* = 0.001] (**Figure 5E**). It is therefore possible that at least part of the activation of PPARγ expression was related to the level of BACE1, which could contribute to suppression of Aβ production.

### Effects of ICS II on Protein Levels of PDE5A in the Hippocampus and Cortex of APP/PS1 Transgenic Mice

**FIGURE 6:**
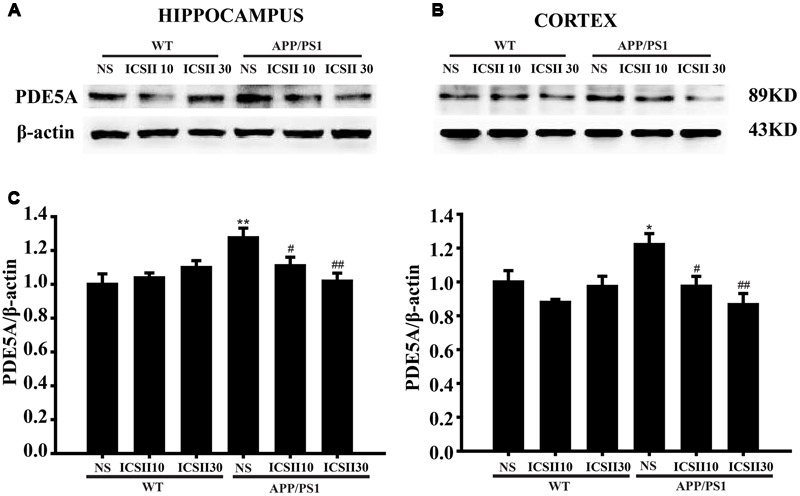
**Effects of ICS II on the protein expression of PDE5A in the hippocampus and cortex of APP/PS1 transgenic mice. (A,B)** The antibody-reactive bands of PDE5A in the hippocampus and cortex of different groups. **(C)** Quantitative analysis of PDE5A protein expression. The relative optical density was normalized to β-actin. Data are presented as mean ± SEM (*n* = 3 each) ^∗^*p* < 0.05, ^∗∗^*p* < 0.01 vs. WT+NS, ^#^*p* < 0.05, ^##^*p* < 0.01 vs. APP/PS1+NS. SEM, standard error of the mean; NS, normal saline; PDE5A, phosphodiesterase type 5A.

The PDE5A level was also measured in the hippocampus and cortex of APP/PS1 transgenic mice by using the PDE51 antibody (*p* < 0.01, *p* < 0.05). Following ICS II treatment for 3 months, a markedly reduced PDE5A level was seen in the hippocampus and cortex with a dose-dependent manner [Hippocampus *F*_(5,12)_ = 4.261, *p* < 0.05 and Cortex *F*_(5,12)_ = 4.806, *p* < 0.05] (*p* < 0.05, *p* < 0.05) (**Figures 6A–C**). This result clearly demonstrated that the overexpression level of PDE5A in hippocampus and cortex of APP/PS1 transgenic mice could be attenuated by ICS II.

## Discussion

This study demonstrated that ICS II protected against spatial learning and memory impairments, decreased senile plaque and Aβ levels, and promoted viable neuron as well as inhibited PDE5 in APP/PS1 transgenic mice.

Although AD is a multifactor disease that the exact pathogenesis is not fully clarity, multiple lines of studies demonstrate that Aβ aggregation in the brain is key factor for the development of AD (Sun et al., 2015; Sengupta et al., 2016). APP/PS1 transgenic model mouse is widely applied to many studies of AD that possesses the overexpression of human APP encoding gene and PS1 gene mutations, and which are related to the familial early-onset AD (Bilkei-Gorzo, 2014; Allue et al., 2016; Choi et al., 2016). In addition, the mouse is not only capable of modeling some main pathological characteristics of AD *via* the excessive accumulation of Aβ, but also the appearance of senile plaque and cognitive dysfunction (Chin, 2011; Chouraki and Seshadri, 2014). Therefore, this study used 12-month-old male APP/PS1 mice to detect the effects of ICS II on cognitive decline and Aβ production. The MWM test results showed substantial spatial learning and memory disorder and neuron loss in APP/PS1 transgenic mice, which are consistent with previous studies (Zhang et al., 2011; Yang R.Y. et al., 2015). Importantly, the deficits were effectively restored by chronic treatment with ICS II.

PDE5 is a 3′, 5′ cyclic guanosine monophosphate hydrolase (Garcia-Osta et al., 2012). Clinically, PDE5-Is are used for treating secondary pulmonary hypertension (Das et al., 2015), and there are recent emerging interests in its role on neurodegenerative diseases (Garcia-Osta et al., 2012). A series of previous evidence indicates that PDE5-Is could improve memory performance in animal models of AD *via* suppressing the levels of Aβ contents (Zhang et al., 2013b) and inhibiting the effect of induced neuronal loss (Yin et al., 2016). It also shows beneficial effects on amyloid deposition (Puzzo et al., 2009). Recently, we found that ICS II as a PDE5-I protects against H_2_O_2_-induced PC12 cells death and attenuates STZ-induced cognitive deficits in rats (Yin et al., 2016; Gao et al., 2017). In this present study, the increased protein expression of PDE5 was found in the brains of APP/PS1 transgenic mice, which is consistent with prior observations (Zhang et al., 2013a; Jin et al., 2014). Thus, we speculated that PDE5 might be involved in the pathogenesis of AD. As expected, ICS II reversed the aberrantly elevated PDE5A protein expression and competed with the cleaved process of APP and further prevented the Aβ production, which finally led to the improvement of the cognitive functions in APP/PS1 transgenic mice. Those results demonstrated that ICS II might work as a PDE5 inhibitor for improving AD symptoms in this mouse model. Lamentedly, it is not clear how ICS II regulates the catalytic activity of PDE5, this effect is necessary to delineate in future experiment.

Extracellular deposition of senile plaque is widely recognized to a critical pathological feature of AD (Joshi et al., 2015) and represents the most significant neuropathological histological feature of the AD (Nilsson et al., 2013). In the AD patients, senile plaques were detected in the hippocampus (Sabri et al., 2015; Witte et al., 2015). Accumulating evidences have shown that 9-month-old APP/PS1 transgenic mice exhibited the formation of senile plaques which gradually increased with age (Bibari et al., 2013; He et al., 2014). Thioflavine S staining results showed that ICS II could efficiently ameliorate the histopathological lesions.

Beta-amyloid peptides are involved in the formation of senile plaques (Sengupta et al., 2016). The most common Aβ peptides consist of 39–43 amino acids. There are two major isoforms of Aβ, soluble Aβ_40_, and insoluble Aβ_42_, but the latter peptide shows more prone to aggregation and stronger neurotoxicity. A series of studies have elucidated elevated levels of Aβ_1-40_ and Aβ_1-42_ in the model mice of AD, which may be the critical factor for causing neuronal deficits and inhibiting the long-term potentiation (LTP) (Zhu et al., 2013; He et al., 2014; Jin et al., 2014). Furthermore, the levels of Aβ contents correlate negatively with the spatial learning and memory functions. Interestingly, the levels of both Aβ contents were potently inhibited in ICS II-treated APP/PS1 mice and the underlying mechanisms are involved in regulating the process of APP, including non-amyloidogenic and amyloidogenic pathways to preclude the generation of Aβ. APP is a precursor of Aβ-peptides (Iakoucheva et al., 2016) and is cleaved by two enzymes (β- and γ-secretases) (Salminen et al., 2013), which are essential steps for generating pathogenic Aβ peptides (a central component of senile plaques in AD brains). It should be noted that the majority of APP is cleaved by α-secretase causing the secretion of sAPPα, an APP extracellular fragment, and the formation of a membrane-bound 83 amino acid fragment (C83). sAPPα exhibits neuroprotective, neurotrophic, and neurogenic properties (Nalivaeva et al., 2014). Moreover, promoting APP α-secretase cleavage is considered as an approach to decrease the Aβ production, in particular because a-secretase appears to compete with BACE1 cleavage site (Saftig and Lichtenthaler, 2015). The balance of α/β-secretases were broken, which leads to non-amyloidogenic cleavage process is gradually interrupted and further facilitates Aβ production in APP/PS1 transgenic mice (He et al., 2014). Several members of the ADAM family, such as ADAM10 and ADAM9 as well as ADAM17, are involved in the activity of α-secretase. Functionally, ADAM10 has neuroprotective and neurotrophic effects, it was defined as the most important member of ADAMs family of α-secretase, which is essential for neurogenesis and development of the embryonic brain (Vassar, 2013; Du et al., 2016). Therefore, promoting ADAM10 protein expression is widely considered to be an effective approach for AD treatment, and the reduced protein expression of ADAM10 has been linked to the molecular pathogenesis of AD (Saftig and Lichtenthaler, 2015). Our results found that inhibition of ADAM10 led to abnormal elevation of Aβ contents *in vivo*. Notably, treatment with ICS II greatly increased ADAM10 expression in the hippocampus and cortex in a concentration dose-dependent manner.

Previous studies have documented that abnormal protein expression of APP and BACE1 could facilitate Aβ production in AD mice and our experiment results agreed with this (Jin et al., 2014; Li et al., 2015; Du et al., 2016). BACE1 is a type I transmembrane aspartic protease that is responsible for cleaving APP at the β-secretase cleavage site to generate sAPPβ and formation of a 99-amino-acid-long C-terminal membrane-bound fragment (C99). In addition, BACE1 undergoes several posttranslational modifications, including phosphorylation, *N*-glycosylation and ubiquitination (Sjölander et al., 2010; Motoki et al., 2012; Vassar et al., 2014). Because of BACE1 prime physiological functions, it has received intensely attention as a promising novel strategy against AD. It was proved that the transcription factor with putative binding sites to be involved in BACE1 expression in AD. eIF2α is an important signaling pathway that affects cognitive functions. The phosphorylation level of eIF2α positively correlated with BACE1 expression level, which can increased the protein level of BACE1 in 5 × FAD mice (Devi and Ohno, 2012, 2013). Similarly, phosphorylated eIF2α at Ser51 are aberrantly elevated associated with the degeneration of neurons in AD patients (Vassar, 2008; Bose et al., 2011). Moreover, PERK as an eIF2α kinase acts on eIF2α phosphorylation site and its phosphatase activity has also been closely related to the increase of BACE1 level (Devi and Ohno, 2014; Yang et al., 2016). In our study, we also found that BACE1, the levels of phosphorylated eIF2α and PERK were significantly higher in APP/PS1 transgenic mice. A progressive enhancement of eIF2α/PERK phosphorylation is responsible for BACE1 overexpression and eventually up-regulation of Aβ production *in vivo* and *in vitro* (Zhu et al., 2013; Zhang et al., 2016). If this were the case, then the fact that chronic treatment of ICS II reversed the induction of PERK-eIF2α pathway may further support the potential utility of ICS II in the treatment of AD. PPARγ is a transcription factor that modulates Aβ metabolism *via* inhibiting BACE1 transcription (Katsouri et al., 2011). Meanwhile, PPARγ signaling pathway is involved in ameliorating AD pathology along with its anti-inflammatory activity. Conversely, activation of the inflammatory factors could reverse activated BACE1 expression and further facilitated Aβ secretion (Katsouri et al., 2011; Strobel et al., 2015). In addition, PPARγ enhancement resulted in suppression of APP expression through promoting ubiquitination of APP and subsequent degradation in SHSY5Y^APP+^ cells (Scuderi et al., 2014). In the present study, we evaluated the expression of PPARγ *in vivo*. Similar to previous findings, we found that PPARγ inhibition could contribute to the elevation of BACE1, which further aggravated APP-Aβ process, and eventually resulted in neurons degradation. Remarkably, ICS II could reduce Aβ production by inducing PPARγ activation, ultimately ameliorating the impairment of learning and memory functions.

**FIGURE 7:**
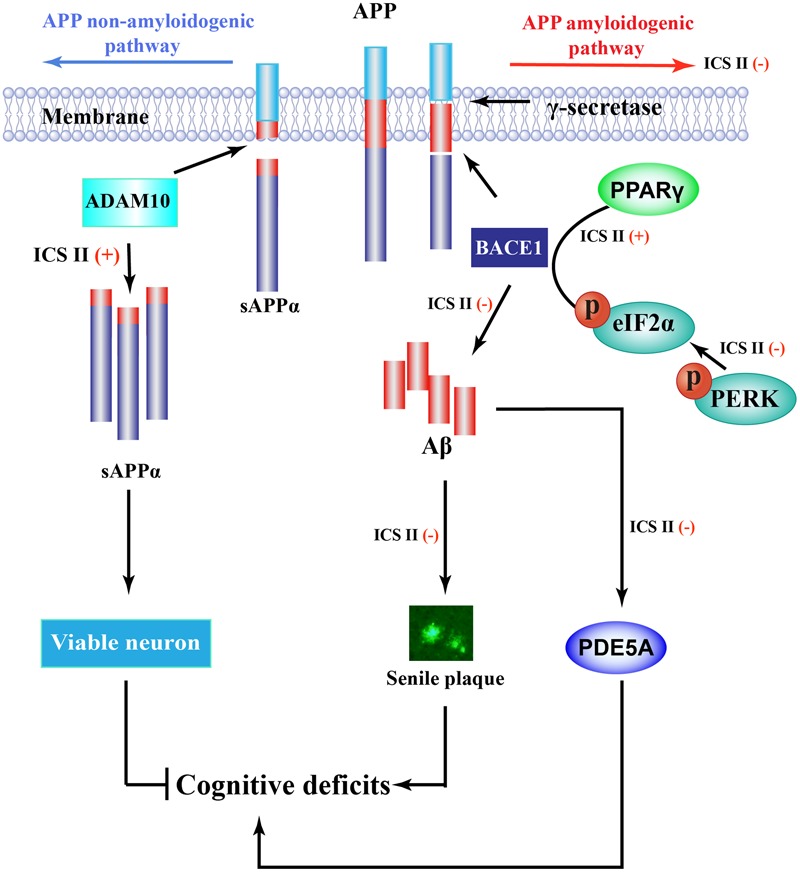
**Overview diagram illustrates the mechanism of ICS II-mediated Aβ production.** Aβ is generated from a sequent cleavage of APP by BACE1 and γ-secretase, ICS II suppressed PPARγ degeneration as well as inhibition of PERK/eIF2α phosphorylation, causing the inhibition of BACE1 and further the inhibition of Aβ production and senile plaque formation. In addition, ICS II prompted non-amyloidogenic cleavage process *via* increasing ADAM10 protein expression, Furthermore, ICS II protected against cognitive deficits may be closely interrelated to inhibition of PDE5.

In summary (**Figure 7**), this study demonstrated that ICS II as a broad spectrum anti-cancer natural compound that remarkably ameliorates learning and memory functions impairment interferes with multiple pathogenic mechanisms, including decreasing the formation of senile plaque, Aβ generation and the neuronal degradation in APP/PS1 transgenic mice. The protective mechanisms are likely attributable to increased ADAM10 activation and suppression of APP and BACE1 expression, these effects appear to be due to the inhibition of PERK/eIF2α/PPARγ signaling pathway. In addition, ICS II protected against cognitive deficits may be closely interrelated to inhibition of PDE5A protein. Combined, the findings provide strong evidence that ICS II may be developed as a potently promising natural compound candidate for halting progression of AD. Nonetheless, the selective PDE5A agonists, BrdU immunohistochemistry and confocal microscopy is under used to further clarify the exact mechanism of ICS II on AD, and that will be as the next chapter in this story.

## Author Contributions

QG and JS: Designed the research; LY and YL: performed the research; FL and JG: analyzed the data; LY: wrote the paper. QG and YD: modified the paper. All authors agreed on the final version of the manuscript.

## Conflict of Interest Statement

The authors declare that the research was conducted in the absence of any commercial or financial relationships that could be construed as a potential conflict of interest.

## Abbreviations

Aβ: beta-amyloid
AD: Alzheimer’s disease
ADAM10: a disintegrin and metalloproteinase domain 10
APP: amyloid precursor protein
APP/PS1: APPswe/PS1dE9
BACE1: β-site APP cleavage enzyme 1
eIF2α: eukaryotic initiation factor α
ICS II: icariside II
MWM: Morris water maze
NFTs: neurofibrillary tangles
NS: normal saline
PBS: phosphate buffer saline
PDE5-Is: phosphodiesterase type 5 inhibitors
PDE5A: phosphodiesterase type 5A
PERK: PKR endoplasmic reticulum regulating kinase
PPARγ: peroxisome proliferator-activated receptor-γ
sAPPα: soluble APP-α
sAPPβ: soluble APP-β
WT: wild type
